# Draft Genome Sequences of Enterohemorrhagic and Enteropathogenic *Escherichia coli* Strains Isolated from Alpacas in Peru

**DOI:** 10.1128/genomeA.01391-17

**Published:** 2018-01-04

**Authors:** Lenin Maturrano, Marjorie Aleman, Dennis Carhuaricra, Jorge Maximiliano, Juan Siuce, Luis Luna, Raul Rosadio

**Affiliations:** a Laboratory of Molecular Biology and Genetics, Faculty of Veterinary Medicine, Universidad Nacional Mayor de San Marcos, Lima, Peru

## Abstract

The draft genome sequences of two strains of *Escherichia coli*, isolated from alpacas in Peru, are reported here. ECA1 has been determined to be a strain of enterohemorrhagic *E. coli* and ECB1 a strain of enteropathogenic *E. coli*. These pathogens are responsible for hemolytic-uremic syndrome in humans and diarrhea in different mammals, respectively.

## GENOME ANNOUNCEMENT

In Peru, alpacas are the principal economic resource of the Andean communities and they suffer from diarrhea associated with pathogenic *Escherichia coli* strains, especially enterohemorrhagic *E. coli* (EHEC) and enteropathogenic *E. coli* (EPEC) strains (1, 2). The gastrointestinal tract of animals, mainly cattle, is considered the most important reservoir of EHEC (3); this pathogen is responsible for serious human diseases, such as hemolytic-uremic syndrome (HUS) (3), which expresses Shiga toxins. EPEC strains are implicated as a cause of diarrhea in humans and in several species of animals (4), and they induce the attaching and effacing (A/E) lesions in the intestinal mucosa, which are mediated by the *eae* gene encoding an outer membrane protein called intimin (5).

In this report, we present the draft genome sequence of EHEC strain ECA1 and EPEC strain ECB1 isolated from alpacas in Peru on a farm in which shedding had been observed (6). Using PCR, strain ECA1 was confirmed to carry one of the virulence genes of EHEC, *stx*_*1*_, encoding Shiga toxin (responsible for HUS), and strain ECB1 was confirmed to carry one of the key genes of EPEC, *eae*. Additionally, the *in silico* serotypes were obtained using SerotypeFinder (7) and determined to be O26:H11 and O76:H2 for ECA1 and ECB1, respectively.

Genomic DNA was isolated from an overnight culture in Luria-Bertani medium using the GeneJET genomic DNA purification kit (Thermo Fisher Scientific). Whole-genome sequencing was performed at Macrogen (Seoul, South Korea). An Illumina HiSeq platform was used to sequence the genomes using a paired-end read length of 2 × 101 bp, and genome libraries were constructed using a TruSeq DNA PCR-free library preparation kit (Illumina, Inc.). The quality of the raw data was checked with FastQC (8), and adapters were trimmed with Trimmomatic (9). The genomes were assembled with Velvet version 1.2.10 (10) and annotated with the NCBI Prokaryotic Genome Annotation Pipeline (11).

The EHEC strain ECA1 and EPEC strain ECB1 draft genomes were determined to be 5,341,965 and 4,928,478 bp in length, distributed as 247 and 179 contigs, with G+C contents of 50.4% and 50.6%, respectively. The genome of ECA1 contains a total of 5,782 genes, including 5,464 coding genes, 6 rRNAs, and 90 tRNAs. The genome of ECB1 contains a total of 5,307 genes, including 4,911 coding genes, 5 rRNAs, and 79 tRNAs.

### Accession number(s).

These whole-genome shotgun projects have been deposited in DDBJ/ENA/GenBank under the accession numbers PEQH00000000 (ECA1) and PEQI00000000 (ECB1). The versions described here are the first versions.
